# Cord blood full blood count parameters in Lagos, Nigeria

**DOI:** 10.11604/pamj.2014.17.192.3680

**Published:** 2014-03-13

**Authors:** Adediran Adewumi, Adeyemo Titilope A, Akinbami A Akinsegun, Gbadegesin Abidoye, Uche Ebele, Akanmu A Sulaimon

**Affiliations:** 1 Department of Haematology and Blood Transfusion, College of Medicine, University of Lagos; 2 Department of Obstetrics and Gynaecology, Lagos State University College of Medicine, Ikeja, Nigeria; 3 Department of Haematology and Blood Transfusion, Lagos State University College of Medicine, Ikeja, Nigeria

**Keywords:** Haemoglobin, cord blood, stem cell, umbilical cord, neonatologist

## Abstract

**Introduction:**

Full blood count (FBC), one of the most frequently requested for laboratory investigations, is a simple, fast and cheap test and is a reliable indicator of health. Due to its usefulness in the assessment of health status of individuals, its parameters in cord blood, a major source of haemopoietic stem cell transplantation and an ideal source for laboratory investigations for newborns were determined to provide a useful guide to local neonatologists and stem cell transplant physicians.

**Methods:**

Three millilitres of umbilical cord blood was collected from 130 normal birth weight newborns (69 males and 61 females) whose cord were clamped immediately after delivery, at a teaching hospital in Lagos, Nigeria and full blood count parameters were determined using Sysmex autoanalyzer, model KX-21N. Consented mothers of the newborns were selected based on, age between 18 and 45 years; uneventful pregnancy and delivery and haemoglobin (Hb) concentration ≥ 10 g/dL.

**Results:**

There were no statistical gender differences in the mean values of Hb concentrations (M = 13.27 ±1.60 g/dL; F = 13.32±1.61g/dL; p = 0.93), total white cell count (M = 3.16±5.43 × 10^9^/L; F = 13.07±4.98 × 10^9^/L; p= 0.92), platelet count (M= 223.64± 64.21 × 10^9^/L; F = 226.69±80.83 × 10^9^/L; p = 0.81) and other parameters.

**Conclusion:**

Mean values of full blood count parameters obtained in this study are comparable to reports from other studies in developing countries and could be a useful guide for neonatologists and stem cell transplant physicians in our geographical location.

## Introduction

A full blood count (FBC) is one of the most frequently requested for investigations in the assessment of health status of an individual. This is because it is a simple, fast and cheap test to obtain and is a reliable indicator of health.

Haemoglobin and haematocrit are important measurements in the diagnosis and treatment of anaemia and polycythaemia [1] while red cell indices provide information about the hemoglobin content and size of red blood cells which are useful in elucidating the etiology of anaemias [1]. RDW is the coefficient of variation of the mean corpuscular volume (MCV) and therefore higher RDW values reflect greater heterogeneity in MCV. This is usually caused by perturbation in erythrocyte maturation or degradation [2]. Furthermore, it is traditionally useful in the differential diagnosis of anaemia [3]. White blood cells and platelets are useful in the assessment of sepsis and haemostatic status respectively.

It is believed that FBC parameters vary with age, sex and race [4]. The results obtained must therefore, be interpreted accordingly. Expectedly, FBC parameters of newborns differ from that of adults in many reports available [5].

Though still farfetched in Nigeria, umbilical cord blood has emerged as a viable source of hematopoietic stem cell transplantation (HSCT) in many other countries [6]. With numerous advantages, umbilical cord blood transplantation (UCBT) has extended the availability of HSCT in the absence of a suitable donor and can be used in urgent situations such as graft failure [7].

The aim of this study was to establish mean values of FBC parameters in Lagos, Nigeria and to compare the mean values with few other reports. Though factors listed above can affect the FBC results, to make clinical decisions, we often rely on European and American populations for our reference values. It is therefore hoped that this report will provide useful information to neonatologist and haemopoietic stem cell transplant (HSCT) physicians in our environment and assist them in their practice.

## Methods

Full blood count parameters (Hb concentration, PCV, red blood cell count, MCV, MCH, MCHC, RDW, WBC and platelet count) in cord blood of 130 full term newborns (gestational age 37- 42 weeks and birth weight of 2.5 - 4kg) were measured by Sysmex autoanalyzer model KX-21N made by Sysmex Coorporation, Kobe, Japan on the same day of collection. The study was carried out at the Maternity Centre of Lagos State University Teaching Hospital after obtaining an ethical approval from hospital Health Research and Ethics Committee and an informed consent from the mothers of the newborns with events free pregnancy, who had vaginal deliveries and whose ages ranged from 18 years to 45 years and haemoglobin concentrations of ≥ 10 g/dL. Excluded from the study were newborns of mothers with multiple pregnancy, eclampsia, diabetes mellitus, HIV infection, chronic diseases such as liver, heart, kidney, lung and those who were delivered by caesarian section. Newborns with birth asphyxia (Apgar score <8 at 5 minutes) and congenital abnormality were also excluded from the study.

**Data analysis**: Analyses were performed using SPSS, version 16. The descriptive data were expressed as mean±S.D. A probability value of p < 0.05 was considered to indicate statistical significance. Pearson Chi square was used for analytical assessment.

## Results

Sixty- nine males and 61 females’ newborns were enrolled in this cross-sectional study between June 2009 and February 2010. The mean values for these parameters for males and females summarized in Table 1 showed no statistical gender difference. The haemoglobin concentration were 13.27 ± 1.60g/dL and 13.32 ± 1.61g/dL (p = 0.86); the white cell count were 13.16 ± 5.43×10^9^/L and 13.07±4.98 × 10^9^/L (p= 0.92) while platelets counts were 223.64 ± 64.21x 109/L and 226.69 ± 80.83× 10^9^/L for males and females respectively. The mean values for red cell indices and red cell distribution widths were also not significantly different. (p values: MCV= 0.72; MCH= 0.99; MCHC= 0.84 and RDW= 0.86).

**Table 1:** Gender distribution of mean values of full blood count parameters in cord blood

Parameter	Male (n = 69)	female (n = 61)	*p* value
Haemoglobin (g/dL)	13.27 ± 1.60	13.32 ±1.61	0.86
PCV (%)	44.75 ± 6.05	44.85 ± 5.51	0.93
Red cell count (x 10^12^/L)	4.09 ± 0.51	4.05 ± 0.6	0.69
MCV (fl)	109.54 ± 11.92	111.29 ± 11.86	0.72
MCH (pg)	32.61 ± 4.13	32.71 ± 4.93	0.99
MCHC (g/dL)	29.79 ±1.67	29.73 ± 1.61	0.84
RDW	19.57 ± 3.82	19.96 ± 4.74	0.86
WBC (x 10^9^/L)	13.16 ± 5.43	13.07 ± 4.98	0.92
Platelets (x10^9^/L)	223.64 ± 64.21	226.69 ± 80.83	0.81

Table 2 compares the mean values of the FBC parameters of this study with other studies from Iraq [8], Pakistan [4], Greece [9] and Taiwan [10]. The mean values obtained in this study were almost similar or close to those from Pakistan [4] and Iraq [8]. For example the mean Hb concentration of our study was 13.29±1.5g/dL and that of Iraq and Pakistan were 13.76 ± 1.46g/dL and 14.99 ± 1.47g/dL respectively; the red cell count in our study was 4.07±0.55 ×10^12^/L, that of Iraq was 4.0 ± 0.47 × 10^12^/L and Pakistan, 4.29 ± 0.44 × 10^12^/L; The mean platelet counts for our study, Iraq and Pakistan were 225.07± 72.21 × 10^9^/L; 267.63 ± 0.62 × 10^9^/L and 256.25 ±76.54 × 10^9^/L respectively. However the mean total WBC count of our study (13.10 ± 5.20 × 10^9^/L), though similar to that of Pakistan (13.61 ± 4.25 × 10^9^/L), was higher than the value from Iraq (10.12 ± 2.8 × 10^9^/L). The mean values of Hb, RCC and WBC of our study were insignificantly higher than reports from Greece [9] and Taiwan [10]. The mean Hb concentration of our study (13.29 ± 1.5g/dL) was higher than that of Greece (8.8 ± 2.9g/dL) and Taiwan (10.9 ± 1.g/dL); the mean RCC of our study (4.07 ± 0.55 × 10^12^/L) was also higher than that of Greece (2.46 ± 0.82 × 10^12^/L) and Taiwan (3.14 ± 0.41 x 1012/L). The values of our red cell indices were not essentially different from that of Greece and Taiwan.

**Table 2:** Comparison of overall mean values ± SD of full blood count parameters in cord blood of this study(n = 130) with other studies

Parameter	Current study (n = 130)	Iraq (n = 220)	Pakistan (n = 404)	Greece (n = 2000)	Taiwan (n = 5602)
Hb (g/dL)	13.29 ± 1.5	13.76 ±1.46	14.99 ± 1.47	8.8 ±2.9	10.9 ± 1.4
PCV (%)	44.8± 5.78	44.42 ± 4.74	45.65 ±4.83	25.9 ± 8.8	36.2 ± 4.3
RCC(x 10^12^/L)	4.07± 0.55	4.0 ± 0.47	4.29 ± 0.44	2.46 ± 0.82	3.14 ± 0.41
MCV (fl)	110.36± 11.88	111.56 ± 6.09	105.81 ± 6.24	105.0 ± 6.0	115.8 ± 6.8
MCH (pg)	32.61± 4.13	34.41 ± 2.36	34.96 ±2.11	35.8 ±	34.8 ± 1.8
MCHC (g/dL	29.76 ± 1.64	30.93 ±1.90	32.47 ± 2.12	34.3 ±7.3	30.1 ±1.2
RDW	19.75 ± 4.26	17.01 ± 1.63	-	12.1 ± 1.6	-
WBC(x 10^9^/L)	13.10 ± 5.20	10.12 ±2.8	13.61 ± 4.23	7.2 ± 3.4	10.0 ± 2.9
Platelets (x10^3^/L)	225.07 ±72.21	267.63 ±60.62	256.25 ±76.54	275 ± 125	217 ± 45

The Comparison of mean values of FBC parameters and gender differences of the parameters with reference range of values published by Dacie and Lewis [11] is presented in Table 3. Insignificant differences were found between few parameters of our report and the reference values published by Dacie and Lewis [11]. In males, the mean values of Hb concentration (13.27± 1.6g/dL) and red cell count (4.09 ± 0.5 × 10^9^/L) were lower than that of reports by Dacie and Lewis [11] (Hb: 15.0 ± 2.0g/dL; RCC- 5.0 ± 0.5 × 10^12^/L). However, the MCV (M: 109.54 ± 11.92 fL; F: 111.29 ± 11.86fL), MCH (M: 32.61 ± 4.13pg; F: 32.71± 4.93pg) and RDW (M:19.57± 3.8; F: 19.96 ± 4.74) in our study were higher than values based on Dacie and Lewis [11] (MCV:M/F-92 ± 9.0fL; MCH: M/F- 29.5 ± 2.5pg; RDW: M/F- 12.8 ± 1.2). Finally, the MCHC (M: 29.79± 1.67g/dL; F: 29.73± 1.61g/dL) and platelet count (M: 223.64 ± 64.21 × 10^9^/L; F: 226.69 ± 80.83 × 10^9^/L) of both sexes were lower than the reference values published by Dacie and Lewis [11] (MCHC: M/F-33.0 ± 1.5g/dL; Platelet count: 275± 125 × 10^9^/L).

**Table 3:** Comparison of mean values ± SD of FBC parameters and gender differences of the parameters with normal range of values based on Dacie and Lewis

Current study	Dacie and Lewis			
Parameter	Male	female	Male	female
Haemoglobin (g/dL)	13.27 ± 1.60	13.32 ±1.61	15.0 ± 2	13.5±1.5
PCV (%)	44.75 ± 6.05	44.85 ± 5.51	45.0 ± 5	41.0 ± 5
Red cell count (x 10^12^/L)	4.09 ± 0.51	4.05 ± 0.6	5.0 ± 0.5	4.3 ± 0.5
MCV (fl)	109.54 ± 11.92	111.29 ± 11.86	92.0 ± 9	92.0 ± 9
MCH (pg)	32.61 ± 4.13	32.71 ± 4.93	29.5 ± 2.5	29.5± 2.5
MCHC (g/dL)	29.79 ±1.67	29.73 ± 1.61	33.0 ± 1.5	33.0 ± 1.5
RDW	19.57 ± 3.82	19.96 ± 4.74	12.8 ± 1.2	12.8 ± 1.2
WBC (x 10^9^/L)	13.16 ± 5.43	13.07 ± 4.98	7.5 ± 3.5	7.5 ± 3.5
Platelets (x10^9^/L)	223.64 ± 64.21	226.69 ± 80.83	275 ± 125	275 ± 125

## Discussion

Cord blood is an ideal source for laboratory examinations for newborns. It reveals the degree of haemopoiesis during foetal life and the clinical conditions such as perinatal asphyxia, meconium staining, chorion amnionitis etc newborns had been subjected to during perinatal period [12]. Blood from umbilical cord is also a rich source of haemopoietic stem cell transplantation [13]. Being easier to obtain and less likely to evoke tissue rejection and transmit infectious agents [14], many have suggested that transplant of cord blood is a viable alternative to bone marrow transplantation for the treatment of a number of genetic disorders and certain cancers. There is therefore a growing need for neonatologists and transplant physicians to compare cord blood test results with reference values. For determination of reference values in a population, minimum sample size of 120 is recommended [15]. However a sample size of 130 was considered for our study to increase precision.

It has been widely suggested that factors such as gestational age, mode of delivery, environment, time of sampling and sex influence FBC results in newborns [16, 17]. However, as shown in Table 1, we did not find a significant gender difference in all the FBC parameters. This corroborates with some other studies that reported that haematologic reference values do not relate to sex [8]. It is however at variance with reference values published by Dacie and Lewis [11] in which the mean values of Hb concentration and red cell count in males were higher in their reports than ours. Other factors such as gestational age, mode of delivery and environmental factors were not considered because all the newborns studied were delivered per vagina, at term and were exposed to the same environmental factors.

Though the values of FBC parameters obtained in this study were almost similar to reports from Iraq, and that of Al-Marzoki et al from Pakistan, they varied from reports from Greece and Taiwan with much larger sample size. The mean values of Hb, RCC and WBC of our study were higher than reports from Greece and Taiwan; while the RDW of our study was higher than that of the report from Greece. The similarity of our reports with that of Iraq and Pakistan appears to disagree with belief that race and geographical locations have major influences of reference values [17]. It may well be that socio-economic factors are major determinants.

Our values also vary slightly with reference values published by Dacie and Lewis [11] in that the MCV, MCH and RDW of both sexes in our study were higher while MCHC and platelet count of both sexes in our study were lower than the reference values published by Dacie and Lewis [11].

We did not find polycythaemia in our study. This may probably be because full term and normal for gestational age newborns were selected for the study since polycythaemia (PCV < 65%) is commonly seen in preterm and small for gestational age (SGA) newborns [17].

## Conclusion

Mean values of full blood count parameters obtained in this study are comparable to reports from other studies in developing countries and could be a useful guide for neonatologists and stem cell transplant physicians in our geographical location.

## References

[1] Mamoury GH, Hamedy AB, Alkhaghi F (2003). Cord haemoglobin in newborn in correlation with maternal haemoglobin in Northeastern Iran. Iran J med Sci..

[2] Kushang V Patel, Luigi Ferrucci, William B Ershler, Dan L Longo, Jack M Guralnik (2009). Red Cell Distribution Width and the Risk of Death in Middle-aged and Older Adults. Arch Intern Med.

[3] Sategna Guidetti C, Scaglione N, Martini S (2002). Red cell distribution width as a marker of coeliac disease: a prospective study. Eur J Gastroenterol Hepatol..

[4] Danish Hassan Qaiser, Syed Tousif Ahmed, Mohammed Perwaiz Sandila, Tahseen Kazmi (2009). Haematological reference values for full term, healthy, newborns of Karachi. http://jpma.org.pk/full_article_text.php?article_id=1800.

[5] Noguera NI, Detarsio G, Perez SM, Bragos IM, Lanza O, Rodriguez JH (1999). Hematologic study of newborn umbilical cord blood. Medicina (B Aires).

[6] O'Brien TA, Tiedemann K, Vowels MR (2006). No longer a biological waste product: Umbilical cord blood. Med J Aust..

[7] Fernandes J, Rocha V, Robin M (2007). Second transplant with two unrelated cord blood units for early graft failure after haematopoietic stem cell transplantation. Br J Haematol..

[8] Al-Marzaki Jasim M, Al-maaroof Zainab W, Kadum Ali H (2012). Determination of reference range for FBC parameters in neonatal cord plasma in Hilla, Babil, Iraq. Journal of Blood Medicine.

[9] Vassilios Katsares, Zissis Paparidis, Eleni Nikolaidou, Ilyana Karvounidou (2009). Reference Ranges for Umbilical Cord Blood Hematological Values. LabMedicine.

[10] Yu-Hsun Chang, Shang-Hsien Yang, Tso-Fu Wang (2011). Complete Blood Count Reference values of Cord Blood in Taiwan and Influence of Gender and Delivery Route on them. Pediatrics & Neonatology.

[11] Dacie JV, Lewis SM (2001). Practical haematology.

[12] Lee JC, Ahern TP, Chaves FP, Quillen K (2010). Utility of haematologic and volume, conductivity and scatter parameters from umbilical cord blood in predicting chorioamnionitis. Int J Lab Haematol..

[13] Benito AI, Diaz MA, Gonzalez-Vicent M (2004). Haemopoietic stem cell transplatation using umbilical cord blood progenitors. Review of current result. Bone Marrow Transplantation..

[14] Rocha V, Wagner JE, Sobocinski KA (2000). Graft versus host disease in children who have received cord blood or bone marrow transplantation from an HLA identical sibling. Engl J Med..

[15] Solberg E, Burtis CA, Ashwood ER, Bruns DE (2006). Establishment and use of reference. Tietz textbook of clinical chemistry and molecular diagnostics.

[16] Ozyure E, Cetintas S, Ceylan T (2006). Complete blood count parameters for healthy, small for gestational age full term newborns. Clinical laboratory haematology..

[17] Keramati MR, Maybodi NT (2013). The effect of Iron deficiency anaemia on the HbA2 level and comparison of haematological values between iron deficiency anaemia and thalassaemia minor. UHOD..

